# Trends in the global, regional, and national burden of pancreatitis among children and adolescents based on the GBD 2021

**DOI:** 10.1097/JS9.0000000000004652

**Published:** 2026-01-13

**Authors:** Mengran Yu, Weiguo Dong

**Affiliations:** Department of Gastroenterology, Renmin Hospital of Wuhan University, Wuhan, Hubei Province, China

**Keywords:** pancreatitis, children and adolescents, disease burden

## Abstract

**Background:**

Increasing pancreatitis incidence among children and adolescents has emerged as a public health challenge. Using data from the Global Burden of Disease (GBD) Study 2021, this study aims to analyze the global, regional, and national burden of pancreatitis in pediatric populations from 1990 to 2021.

**Methods:**

Using GBD 2021 data, we analyzed numbers and age-standardized rates of incidence, mortality, and disability-adjusted life years (DALYs) in populations aged 0–19 years at global, regional, and national levels, stratified by sex, age, and sociodemographic index (SDI). Furthermore, decomposition analysis, frontier analysis, and Bayesian age-period-cohort (BAPC) prediction were performed.

**Results:**

Globally, the incidence burden of pancreatitis in children and adolescents increased from 1990 to 2021, with the number of cases rising from 156 383.64 to 194 636.05 and the age-standardized incidence rate (ASIR) increasing from 6.91 to 7.18 per 100 000. Conversely, mortality and DALY burdens declined, with deaths decreasing from 1309.23 to 1120.09 and the age-standardized mortality rate (ASMR) dropping from 0.06 to 0.04 per 100 000. DALY cases decreased from 113 917.02 to 100 041.07 and the age-standardized DALY rate (ASDR) dropped from 4.99 to 3.65 per 100 000. Adolescents aged 15–19 years demonstrated the highest disease burden. Females exhibited higher incidence, while males demonstrated significantly higher mortality and DALYs. Predictions indicate sustained reductions in ASIR, ASMR, and ASDR until 2040.

**Conclusion:**

Despite declining mortality and DALY burdens, pancreatitis incidence among children and adolescents has increased, underscoring its significance as a global health threat. Geographic, age, gender, and SDI disparities persist.

## Introduction

Pancreatitis, an inflammatory disorder of the pancreas, is categorized into three distinct clinical classifications: acute pancreatitis (AP), acute recurrent pancreatitis (ARP), and chronic pancreatitis (CP). Historically considered an uncommon clinical entity in pediatric populations, limited epidemiological studies have revealed a rising incidence, attracting more attention than before. For example, an analysis covering 2007–2014 reported an incidence of 6.2–7.1 cases per 100 000 based on Truven MarketScan Research Databases^[1]^, and a study in Cincinnati spanning 2010–2015 documented rates of 6.1–8.8 cases per 100 000^[2]^.

**HIGHLIGHTS**

- Both incidence cases and age-standardized incidence rate of pancreatitis in children and adolescents increased.
- Both the numbers and the age-standardized rates of deaths and disability-adjusted life years decreased.
- Low-middle and low sociodemographic index regions, Eastern Europe, and Andean Latin America, 15–19 year-old adolescents, and males experienced a higher burden of pediatric pancreatitis than their counterparts.

Unlike adults, severe complications such as pancreatic necrosis or multi-organ dysfunction are infrequent in children and adolescents, affecting fewer than 6% of cases^[3,4]^. However, approximately 15%–35% of pediatric AP episodes progress to ARP or CP^[4,5]^, resulting in long-term sequelae including diabetes, malnutrition, and an increased risk of pancreatic cancer^[6]^. Furthermore, the economic burden of pediatric pancreatitis is substantial, with annual hospitalization costs about $200 million in the United States, excluding the intensive care and surgical interventions of complex pancreatitis, as well as indirect societal impacts such as caregiver absenteeism and missed school days^[3]^. Concurrently, CP significantly impairs health-related quality of life (HRQOL), particularly in physical functionality, social engagement, and academic performance^[7]^. Persistent abdominal pain and lower socioeconomic status are key determinants of impaired HRQOL, affecting physical and psychological health and highlighting the need for holistic management strategies^[8–11]^.

Pediatric pancreatitis exhibits distinct etiological profiles, clinical manifestations, and prognostic outcomes compared to adult pancreatitis. While advancements in diagnostic modalities and clinical awareness have increased recognition of pediatric cases, epidemiological insights remain fragmented. Most existing studies have temporal and geographical limitations, with predominant evidence derived from historical cohorts primarily from the United States; therefore, global epidemiological patterns remain inadequately characterized^[1,2,12]^. To bridge these knowledge gaps, our study assessed pancreatitis incidence, mortality, and disability-adjusted life years (DALYs) burden in children and adolescents (aged 0–19 years) at global, regional, and national levels, stratified by sex, age, and sociodemographic index (SDI). We also conducted decomposition analysis, frontier analysis, and Bayesian age-period-cohort (BAPC) predictions. This research aims to fill a critical evidence gap, offer policymakers insights to prioritize resource allocation and guide targeted prevention strategies, and advocate for enhanced international attention to this emerging pediatric health challenge.

This study has been reported in line with the STROCSS guidelines^[13]^.

## Methods

### Data source

All data used in this study were derived from the Global Burden of Disease (GBD) 2021 database, managed by the Institute for Health Metrics and Evaluation (IHME). GBD 2021 is a systematically compiled and rigorously validated resource that provides comprehensive estimates of incidence, mortality, DALYs, and risk factors for 371 diseases and injuries across 21 GBD regions and 204 countries/territories from 1990 to 2021. The data were extracted from the GBD results, publicly accessible at https://vizhub.healthdata.org/gbd-results/. Pancreatitis was coded as DC31, DC32, DC33, and DC34 according to the 11th version of the International Classification of Diseases (ICD), available at: https://icd.who.int/browse/2025-01/mms/en. According to the World Health Organization definition, children and adolescents refer to individuals aged 0–19 years, which constitutes the study population in our research.

SDI is a composite metric designed to characterize the relationship between socioeconomic development and health outcomes, composed of three key components: educational attainment, per capita income, and total fertility rate^[14]^. Its value ranges from 0 to 1, where values approaching 1 indicate higher levels of socioeconomic development, while values closer to 0 reflect lower development status. Based on SDI values, countries/territories are categorized into five distinct tiers: high SDI (>0.81), high-middle SDI (0.70–0.81), middle SDI (0.61–0.69), low-middle SDI (0.46–0.60), and low SDI (< 0.46)^[15]^.

### Statistical analysis

The disease burden was quantified through three principal indicators: incidence, mortality, and DALYs, and 95% uncertainty intervals (UIs) were reported to reflect variability in estimates. To address variations in population age structures across geographic regions and temporal periods, we employed age-standardized incidence rate (ASIR), age-standardized mortality rate (ASMR), and age-standardized DALY rate (ASDR) based on the GBD reference population. The calculation formula of age-standardized rate (ASR) is $\frac{\sum_{i=1}^{A}aiwi}{\sum_{i}^{A}wi}$, where *a_i_* represents the age-specific rate for the *i*^*th*^ age group, *w_i_* denotes the corresponding weight from age subgroup of the chosen reference standard population, and *A* is the total number of age groups.

Estimated annual percentage change (EAPC) with its corresponding 95% confidence interval (CI), obtained from a log-linear regression model, was employed to reveal temporal changes in ASIR, ASMR, and ASDR over time. An upward trend was defined when both the EAPC and the lower bound of its 95% CI exceeded 0; a downward trend was defined when both the EAPC and the upper bound of its 95% CI fell below 0; and stability was defined when the 95% CI of the EAPC encompassed 0.

Fitted curves were employed to examine the association between pancreatitis disease burden and SDI. Decomposition analysis was conducted to quantify the absolute and relative impacts of population growth, population aging, and epidemiological changes to variations in incidence and DALYs between 1990 and 2021. Frontier analysis was implemented via nonparametric data envelopment analysis to identify the theoretical minimum achievable disease burden for each SDI level. Bootstrap resampling (1000 iterations) was performed to ensure robust estimation of frontier CIs. The BAPC model was employed to project the disease burden up to 2040. All analyzes were executed using R statistical software (version.4.4.2, R Foundation for Statistical Computing). Statistical significance was defined as *P* < 0.05 for all hypothesis tests.

The work has been reported in line with the STROCSS criteria.

## Results

### Global level

**Figure 1.**
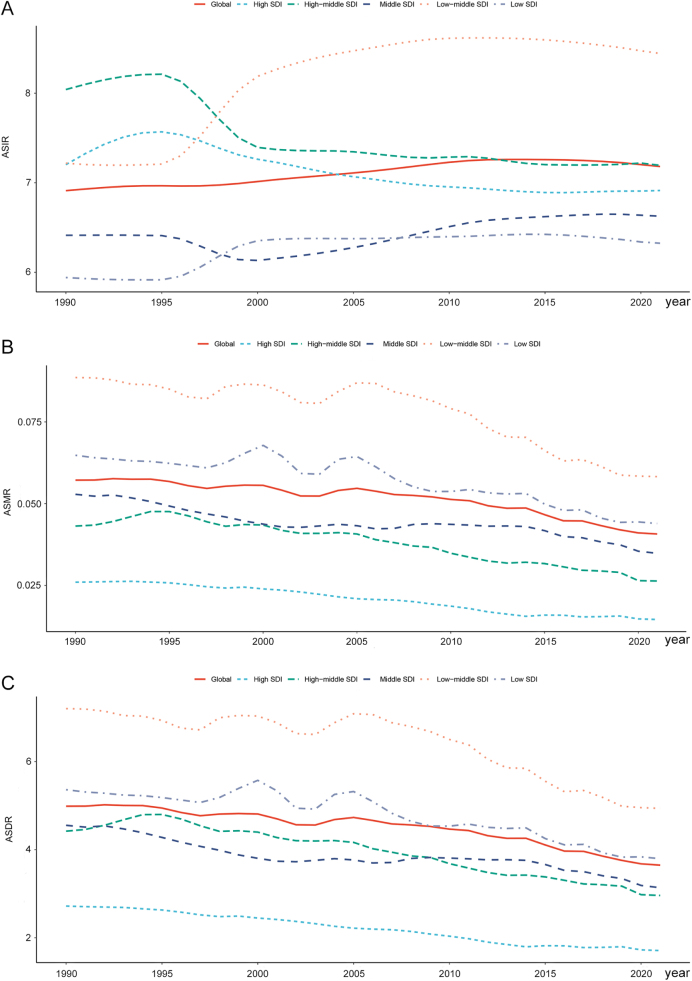
Trends in ASIR (A), ASMR (B), and ASDR (C) for pancreatitis among children and adolescents (0–19 years) from 1990 to 2021 across different SDI levels. ASIR, age-standardized incidence rate; ASMR, age-standardized mortality rate; ASDR, age-standardized disability-adjusted life-year rate; SDI, sociodemographic index.

Overall, pancreatitis remains a significant health threat for children and adolescents. The number of incidence cases worldwide increased from 156 383.64 (95% UI: 93 275.99–244 084.37) in 1990 to 194 636.05 (95% UI: 119 106.26–298 345.61) in 2021, with an ASIR of 6.91/100 000 (95% UI: 4.12–10.80) in 1990 and 7.18/100 000 (95% UI: 4.39–11.02) in 2021, respectively. This rate rose at an average of 0.18 (95% CI: 0.15–0.20) per year from 1990 to 2021 (EAPC). However, the mortality and DALYs both showed a downward trend. The death cases decreased from 1309.23 (95% UI: 1009.04–1871.52) in 1990 to 1120.09 (95% UI: 892.98–1422.26) in 2021, and DALY cases declined from 113917.02 (95% UI: 88747.43–159344.57) in 1990 to 10 0041.07 (95% UI: 79 627.49–126 728.92) in 2021. The ASMR was low, at 0.06/100 000 (95% UI: 0.04–0.08) and 0.04/100 000 (95% UI: 0.03–0.05) in 1990 and 2021, respectively, reflecting a downward trend [EAPC: −1.04 (95% CI: −1.19 to −0.89)]. At the same time, the ASDR fell from 4.99/100 000 (95% UI: 3.88–6.98) to 3.65/100 000 (95% UI: 2.90–4.63) from 1990 to 2021, with an EAPC of −0.96 (95% CI: −1.08 to −0.84; Table 1, Fig. 1).

**Table 1:** Incidence, mortality, and DALYs of pancreatitis among children and adolescents between 1990 and 2021 at the global and regional levels.

	Incidence					Mortality					DALYs				
	Cases, 1990	Cases, 2021	ASIR (per 100 000), 1990	ASIR (per 100 000), 2021	EAPC, 1990–2021	Cases, 1990	Cases, 2021	ASMR (per 100 000), 1990	ASMR (per 100 000), 2021	EAPC, 1990–2021	Cases, 1990	Cases, 2021	ASDR (per 100 000), 1990	ASDR (per 100 000), 2021	EAPC, 1990–2021
Global	156 383.64 (93 275.99, 244 084.37)	194 636.05 (119 106.26, 298 345.61)	6.91 (4.12, 10.80)	7.18(4.39, 11.02)	0.18 (0.15, 0.20)	1309.23 (1009.04, 1871.52)	1120.09 (892.98, 1422.26)	0.06 (0.04, 0.08)	0.04 (0.03, 0.05)	−1.04 (−1.19, −0.89)	113 917.02 (88 747.43, 159 344.57)	100 041.07 (79 627.49, 126 728.92)	4.99 (3.88, 6.98)	3.65 (2.90, 4.63)	−0.96 (−1.08, − 0.84)
Sex															
Male	77 396.35 (46 206.26, 120 652.37)	95 983.68 (58 636.95, 147 338.08)	6.69 (3.99, 10.44)	6.88(4.20,10.57)	0.15 (0.12, 0.18)	767.19 (571.45, 1129.81)	703.65 (522.39, 907.77)	0.07 (0.05, 0.10)	0.05 (0.04, 0.06)	−0.83 (−0.99, − 0.67)	65 584.06 (49 466.54, 94 058.55)	61 018.58 (45 544.23, 79 267.05)	5.62 (4.24, 8.06)	4.33 (3.22, 5.63)	−0.79 (−0.93, −0.66)
Female	78 987.30 (46 845.59, 123 701.06)	98 652.38 (60 374.98, 150 836.54)	7.15 (4.24, 11.20)	7.50 (4.59, 11.49)	0.20 (0.18, 0.22)	542.05 (350.62, 870.41)	416.44 (284.60, 583.87)	0.05 (0.03, 0.08)	0.03 (0.02, 0.04)	−1.38 (−1.53, −1.23)	48 332.96 (32 929.12, 73 578.37)	39 022.49 (27 964.06, 53 321.33)	4.33 (2.95, 6.60)	2.93 (2.10, 4.01)	−1.22 (−1.34, −1.11)
SDI															
High	19 013.07 (11 349.49, 29 258.69)	17 119.13 (11 948.54, 23 495.30)	7.20 (4.29, 11.13)	6.91 (4.81, 9.53)	−0.31 (−0.37, −0.26)	70.06 (64.26, 76.56)	37.12 (34.67, 39.90)	0.03 (0.02, 0.03)	0.01 (0.01, 0.02)	−2.15 (−2.30, −2.00)	7265.01 (6120.25, 8984.32)	4296.94 (3550.04, 5462.77)	2.72 (2.29, 3.37)	1.71 (1.41, 2.18)	−1.66 (−1.75, −1.58)
High-middle	30 855.75 (18 874.27, 46 923.92)	22 584.64 (14 238.31, 33 105.82)	8.04 (4.91, 12.27)	7.19 (4.54, 10.55)	−0.45 (−0.55, −0.36)	173.05 (143.54, 213.46)	84.38 (76.18, 97.31)	0.04 (0.04, 0.05)	0.03 (0.02, 0.03)	−1.81 (−2.01, −1.61)	17 485.16 (13 898.73, 22 852.21)	9441.16 (7540.95, 12 502.45)	4.42 (3.49, 5.82)	2.96 (2.36, 3.93)	−1.51 (−1.67, −1.36)
Middle	50 069.20 (29 024.82, 78 796.57)	51 778.72 (31 258.08, 80 024.83)	6.41 (3.71, 10.12)	6.63 (4.00, 10.26)	0.19 (0.11, 0.27)	422.98 (331.88, 541.45)	278.57 (231.89, 331.39)	0.05 (0.04, 0.07)	0.03 (0.03, 0.04)	−1.03 (−1.19, −0.86)	36 241.56 (28 808.57, 46 277.32)	24 971.52 (20 681.33, 30 320.40)	4.55 (3.61, 5.84)	3.14 (2.59, 3.82)	−0.92 (−1.07, −0.77)
Low-middle	41 018.66 (24 172.13, 64 965.03)	66 799.14 (39 387.72, 104 437.90)	7.22 (4.26, 11.40)	8.44 (4.97, 13.23)	0.65 (0.50, 0.79)	485.38 (345.09, 775.66)	472.42 (343.33, 635.15)	0.09 (0.06, 0.14)	0.06 (0.04, 0.08)	−1.30 (−1.56, −1.04)	39 706.93 (28 339.34, 61 972.20)	39 856.57 (29 613.61, 52 835.44)	7.20 (5.18, 11.19)	4.94 (3.67, 6.55)	−1.16 (−1.40, −0.93)
Low	15 304.06 (8790.14, 24 448.22)	36 243.30 (21 152.68, 57 776.02)	5.94 (3.42, 9.44)	6.33 (3.69, 10.07)	0.27 (0.19, 0.35)	156.84 (97.45, 280.13)	246.95 (177.24, 340.60)	0.06 (0.04, 0.11)	0.04 (0.03, 0.06)	−1.26 (−1.48, −1.04)	13 135.38 (8355.42, 22 696.28)	21 416.04 (15 703.30, 29 164.00)	5.36 (3.52, 9.03)	3.80 (2.79, 5.15)	−1.12 (−1.31, −0.93)
Region															
Andean Latin America	1514.72 (905.23, 2361.59)	1873.11 (1214.64, 2773.48)	8.10 (4.85, 12.62)	7.73 (5.00, 11.46)	−0.27 (−0.33, −0.20)	52.50 (35.37, 75.89)	29.53 (20.93, 41.25)	0.28 (0.19, 0.41)	0.12 (0.09, 0.17)	−2.68 (−2.84, −2.52)	4139.30 (2775.71, 5983.38)	2323.40 (1659.71, 3232.34)	22.11 (14.87, 31.90)	9.54 (6.81, 13.28)	−2.69 (−2.85, −2.52)
Australasia	408.77 (236.96, 650.26)	449.98 (261.20, 707.01)	6.06 (3.49, 9.70)	5.72 (3.32, 9.00)	−0.18 (−0.23, −0.13)	0.72 (0.61, 0.86)	0.36 (0.29, 0.43)	0.01 (0.01, 0.01)	0.00 (0.00, 0.01)	−2.54 (−2.84, −2.24)	87.43 (68.00, 115.49)	62.30 (43.07, 91.77)	1.30 (1.01, 1.72)	0.79 (0.55, 1.17)	−1.44 (−1.62, −1.27)
Caribbean	1059.90 (612.58, 1688.57)	1070.66 (621.06, 1688.49)	6.93 (4.00, 11.07)	6.82 (3.95, 10.78)	−0.04 (−0.06, −0.03)	8.69 (6.05, 12.98)	6.98 (4.48, 10.89)	0.06 (0.04, 0.08)	0.04 (0.03, 0.07)	−0.62 (−0.78, −0.46)	750.94 (529.14, 1124.28)	614.12 (401.10, 933.46)	4.81 (3.37, 7.25)	3.85 (2.49, 5.92)	−0.57 (−0.71, −0.44)
Central Asia	2664.29 (1552.99, 4133.66)	2990.74 (1758.95, 4611.05)	8.68 (5.06, 13.46)	8.87 (5.22, 13.66)	0.02 (0.00, 0.04)	14.92 (10.58, 19.64)	10.08 (8.21, 12.64)	0.05 (0.03, 0.06)	0.03 (0.03, 0.04)	−1.74 (−1.90, −1.58)	1727.62 (1151.93, 2502.74)	1411.32 (979.52, 2133.25)	5.58 (3.75, 8.05)	4.27 (2.98, 6.42)	−1.08 (−1.18, −0.98)
Central Europe	3062.61 (1835.00, 4717.67)	1415.21 (972.07, 1954.34)	7.47 (4.47, 11.52)	5.77 (3.96, 7.99)	−0.76 (−0.87, −0.65)	20.98 (18.60, 23.31)	7.09 (6.28, 7.96)	0.05 (0.04, 0.06)	0.03 (0.02, 0.03)	−2.03 (−2.26, −1.80)	1974.64 (1670.53, 2382.09)	698.70 (584.42, 863.07)	4.77 (4.02, 5.77)	2.76 (2.30, 3.43)	−1.82 (−1.98, −1.66)
Central Latin America	8015.88 (4939.65, 12 177.54)	8762.92 (5570.20, 13 134.09)	9.75 (6.01, 14.81)	9.85 (6.26, 14.81)	0.06 (0.03, 0.09)	85.84 (75.82, 96.00)	79.05 (69.27, 89.36)	0.10 (0.09, 0.12)	0.08 (0.07, 0.10)	−0.39 (−0.69, −0.09)	7122.93 (6225.27, 8024.26)	6455.74 (5667.83, 7354.95)	8.69 (7.60, 9.79)	6.92 (6.06, 7.90)	−0.43 (−0.70, −0.15)
Central Sub-Saharan Africa	1266.05 (693.81, 2096.14)	3132.09 (1704.36, 5153.76)	4.44 (2.44, 7.32)	4.38 (2.39, 7.19)	−0.03 (−0.05, −0.01)	8.58 (4.34, 16.60)	19.33 (11.20, 32.73)	0.03 (0.02, 0.06)	0.03 (0.02, 0.05)	−0.23 (−0.34, −0.12)	755.38 (405.88, 1367.33)	1718.22 (1051.83, 2726.36)	2.80 (1.53, 5.01)	2.50 (1.54, 3.97)	−0.20 (−0.29, −0.10)
East Asia	28 906.87 (15 834.82, 47 310.46)	12 839.92 (7179.14, 21 001.70)	5.98 (3.26, 9.86)	3.66 (2.05, 5.97)	−1.75 (−2.21, −1.28)	233.16 (159.57, 322.66)	58.92 (43.88, 78.85)	0.05 (0.03, 0.06)	0.02 (0.01, 0.02)	−3.29 (−3.38, −3.20)	19 794.52 (14 118.28, 26 913.80)	5277.15 (3979.28, 6883.15)	3.95 (2.78, 5.44)	1.50 (1.13, 1.95)	−3.18 (−3.31, −3.05)
Eastern Europe	11 670.51 (6913.53, 17 898.47)	8820.51 (5272.12, 13 417.33)	17.09 (10.12, 26.20)	18.46 (11.05, 28.08)	0.19 (0.03, 0.36)	39.22 (34.63, 42.90)	30.41 (28.00, 32.61)	0.06 (0.05, 0.06)	0.06 (0.06, 0.07)	−0.11 (−0.65, 0.44)	5650.65 (3988.62, 8516.78)	4231.60 (3043.73, 6276.30)	8.17 (5.75, 12.34)	8.79 (6.34, 13.01)	−0.03 (−0.42, 0.36)
Eastern Sub-Saharan Africa	4892.47 (2732.03, 7942.41)	10 653.06 (5968.93, 17 235.00)	4.75 (2.66, 7.68)	4.74 (2.66, 7.66)	−0.05 (−0.08, −0.02)	24.70 (14.53, 47.32)	52.74 (32.12, 80.25)	0.03 (0.02, 0.05)	0.02 (0.01, 0.04)	−0.14 (−0.17, −0.11)	2356.18 (1424.36, 4208.83)	5024.40 (3236.81, 7301.53)	2.36 (1.47, 4.08)	2.25 (1.45, 3.27)	−0.11 (−0.13, −0.08)
High-income Asia Pacific	5995.89 (3630.58, 9180.18)	3520.90 (2292.43, 5089.66)	11.24 (6.80, 17.30)	10.87 (7.08, 15.74)	−0.39 (−0.47, −0.30)	17.73 (13.44, 23.09)	3.81 (3.39, 4.41)	0.03 (0.02, 0.04)	0.01 (0.01, 0.01)	−3.64 (−4.01, −3.28)	2194.54 (1604.04, 3085.75)	672.80 (479.80, 971.72)	3.96 (2.89, 5.58)	2.03 (1.44, 2.95)	−2.21 (−2.48, −1.95)
High-income North America	5966.50 (3601.85, 9047.57)	7498.35 (5590.24, 9611.98)	7.03 (4.23, 10.70)	7.65 (5.68, 9.85)	0.07 (0.00, 0.14)	21.06 (20.19, 21.96)	19.14 (17.89, 20.70)	0.02 (0.02, 0.03)	0.02 (0.02, 0.02)	−1.28 (−1.55, −1.01)	2074.10 (1814.30, 2471.82)	2048.93 (1742.90, 2528.99)	2.46 (2.15, 2.93)	2.08 (1.76, 2.58)	−0.86 (−1.06, −0.65)
North Africa and Middle East	10 460.12 (6085.03, 16 438.13)	13 958.87 (8213.35, 21 768.70)	6.09 (3.55, 9.55)	5.84 (3.44, 9.11)	−0.05 (−0.08, −0.01)	38.27 (24.53, 58.32)	38.30 (24.99, 53.94)	0.02 (0.01, 0.03)	0.02 (0.01, 0.02)	−0.68 (−0.85, −0.51)	4161.09 (2751.76, 6225.87)	4524.67 (3016.05, 6406.72)	2.42 (1.61, 3.60)	1.90 (1.26, 2.69)	−0.53 (−0.65, −0.41)
Oceania	131.13 (70.95, 218.28)	239.28 (130.26, 391.89)	4.02 (2.18, 6.68)	3.88 (2.11, 6.34)	−0.14 (−0.15, −0.12)	1.99 (0.78, 3.77)	2.62 (1.29, 4.85)	0.06 (0.02, 0.12)	0.04 (0.02, 0.08)	−1.74 (−2.11, −1.38)	163.57 (70.15, 301.15)	219.01 (113.59, 397.59)	5.10 (2.18, 9.38)	3.63 (1.88, 6.57)	−1.66 (−2.00, −1.32)
South Asia	44 278.66 (26 341.05, 69 738.98)	80 723.42 (48 207.88, 125 560.52)	8.50 (5.07, 13.34)	11.04 (6.57, 17.24)	1.01 (0.80, 1.22)	539.47 (373.59, 884.74)	507.87 (350.63, 691.57)	0.11 (0.08, 0.18)	0.07 (0.05, 0.09)	−1.54 (−1.90, −1.18)	43 510.28 (30 622.80, 69 779.06)	42 555.93 (30 294.71, 57 358.65)	8.66 (6.14, 13.83)	5.59 (3.97, 7.55)	−1.37 (−1.70, −1.05)
Southeast Asia	11 027.50 (6212.80, 17 849.76)	11 779.49 (6745.31, 18 649.49)	4.99 (2.81, 8.08)	4.94 (2.82, 7.84)	−0.04 (−0.05, −0.03)	76.35 (50.16, 129.82)	66.26 (48.45, 111.98)	0.03 (0.02, 0.06)	0.03 (0.02, 0.05)	−0.93 (−1.00, −0.86)	6674.58 (4555.62, 10 917.64)	5990.64 (4375.52, 9352.06)	3.03 (2.07, 4.96)	2.44 (1.78, 3.82)	−0.77 (−0.83, −0.71)
Southern Latin America	1246.75 (759.41, 1908.91)	1715.26 (1071.34, 2532.25)	6.36 (3.87, 9.75)	8.21 (5.12, 12.15)	0.28 (0.08, 0.48)	14.55 (12.62, 16.62)	9.54 (8.17, 11.25)	0.07 (0.06, 0.08)	0.04 (0.04, 0.05)	−1.20 (−1.46, −0.93)	1217.80 (1057.30, 1409.97)	856.04 (726.38, 1027.04)	6.19 (5.37, 7.17)	3.97 (3.36, 4.78)	−1.06 (−1.28, −0.84)
Southern Sub-Saharan Africa	1393.77 (792.75, 2223.63)	1642.82 (939.25, 2604.91)	5.33 (3.04, 8.50)	5.17 (2.95, 8.20)	−0.17 (−0.23, −0.11)	3.11 (1.69, 4.93)	5.57 (3.41, 8.13)	0.01 (0.01, 0.02)	0.02 (0.01, 0.03)	1.04 (0.86, 1.21)	381.76 (232.13, 600.40)	588.29 (368.13, 858.59)	1.47 (0.90, 2.32)	1.84 (1.15, 2.68)	0.60 (0.48, 0.71)
Tropical Latin America	1875.26 (1136.13, 2823.74)	1459.33 (861.76, 2211.78)	2.66 (1.61, 4.01)	2.12 (1.25, 3.22)	−0.70 (−0.82, −0.57)	42.70 (38.48, 47.17)	39.07 (35.27, 42.62)	0.06 (0.05, 0.07)	0.05 (0.05, 0.06)	0.59 (0.29, 0.89)	3423.09 (3071.26, 3813.29)	3078.09 (2763.20, 3399.28)	4.88 (4.37, 5.44)	4.34 (3.89, 4.81)	0.51 (0.22, 0.80)
Western Europe	5016.45 (2931.24, 7891.82)	5041.99 (3426.35, 7088.32)	4.74 (2.75, 7.53)	5.17 (3.50, 7.30)	0.16 (0.04, 0.28)	22.51 (21.01, 23.80)	9.02 (8.41, 9.60)	0.02 (0.02, 0.02)	0.01 (0.01, 0.01)	−2.76 (−2.89, −2.62)	2078.98 (1852.26, 2365.63)	1027.89 (864.82, 1262.72)	1.93 (1.71, 2.20)	1.04 (0.87, 1.28)	−2.08 (−2.17, −1.98)
Western Sub-Saharan Africa	5529.56 (3141.95, 8876.72)	15 048.14 (8583.89, 24 115.35)	5.59 (3.18, 8.92)	5.82 (3.32, 9.30)	0.11 (0.09, 0.13)	42.18 (23.70, 70.39)	124.42 (73.70, 182.63)	0.05 (0.03, 0.07)	0.05 (0.03, 0.07)	0.47 (0.39, 0.55)	3677.64 (2145.94, 5992.52)	10 661.84 (6594.13, 15 529.11)	3.88 (2.37, 6.04)	4.27 (2.68, 6.13)	0.43 (0.35, 0.50)

DALY, disability-adjusted life-year; ASIR, age-standardized incidence rate; ASMR, age-standardized mortality rate; ASDR, age-standardized disability-adjusted life-year rate; EAPC, estimated average percent change; SDI, sociodemographic index.

When it comes to gender, the ASIR of females was higher than that of males from 1990 to 2021 (males: from 6.69/100 000 to 6.88/100 000, females: from 7.15/100 000 to 7.50/100 000), and grew faster [male: 0.15 (95% CI: 0.12–0.18), female: 0.20 (95% CI: 0.18–0.22)]. However, males experienced greater burdens of mortality and DALYs than females in 1990 and 2021. To be specific, the global number of death cases caused by pancreatitis was 703.65 (95% UI: 522.39–907.77) in males and 416.44 (95% UI: 284.60–583.87) in females in 2021, with the ASMR of 0.05/100 000 (95% UI: 0.04–0.06) and 0.03/100 000 (95% UI: 0.02–0.04) for males and females, respectively. Both genders saw a decreasing trend in ASMR [male: −0.83 (95% CI: −0.99 to −0.67), female: −1.38 (95% CI: −1.53 to −1.23)]. The ASDR for males was 4.33/100 000 (95% UI: 3.22–5.63) in 2021, which was higher than that of females with 2.93/100 000 (95% UI: 2.10–4.01). Moreover, females experienced a faster annual decline than males [EAPC: male: −0.79 (95% CI: −0.93 to −0.66), female: −1.22 (95% CI: −1.34 to −1.11); Table 1].

### SDI level

When stratified by the SDI, low-middle SDI and middle SDI regions exhibited the greatest disease burden among the five SDI regions in terms of incidence cases [low-middle SDI: 66 799.14 (95% UI: 39 387.72–104 437.90), middle SDI: 51 778.72 (95% UI: 31 258.08–80 024.83)], death cases [low-middle SDI: 472.42 (95% UI: 343.33–635.15), middle SDI: 278.57 (95% UI: 231.89–331.39)], and DALY cases [low-middle SDI: 39 856.57 (95% UI: 29 613.61–52 835.44), middle SDI: 24 971.52 (95% UI: 20 681.33–30 320.40)] in 2021 (Table 1). As for the ASRs, high-middle SDI regions demonstrated the highest ASIR in 1990, followed by a decline after 1995. By 2021, low-middle SDI regions recorded the highest ASIR [8.44/100 000 (95% UI: 4.97–13.23)]. Notably, middle SDI and low SDI regions maintained consistently lower ASIRs in the past 32 years (Table 1, Fig. 1A). Regarding mortality and DALY burden, low-middle SDI regions [ASMR: 0.06/100 000 (95% UI: 0.04–0.08), ASDR: 4.94/100 000 (95% UI: 3.67–6.55)] and low SDI regions [ASMR: 0.04/100 000 (95% UI: 0.03–0.06), ASDR: 3.80/100 000 (95% UI: 2.79 to 5.15)] exhibited higher ASMR and ASDR than the global level from 1990 to 2021, while high, high-middle, and middle SDI regions exhibited lower ASMR and ASDR than the global level from 1990 to 2021. Despite regional disparities, all five SDI regions experienced decreasing trends in ASMR and ASDR over time, with high SDI regions achieving the fastest decline [EAPC of ASMR: −2.15 (95% CI: −2.30 to −2.00), EAPC of ASDR: − 1.66 (95% CI: −1.75 to −1.58); Table 1, Fig. 1B,C].

### Regional level

**Figure 2.**
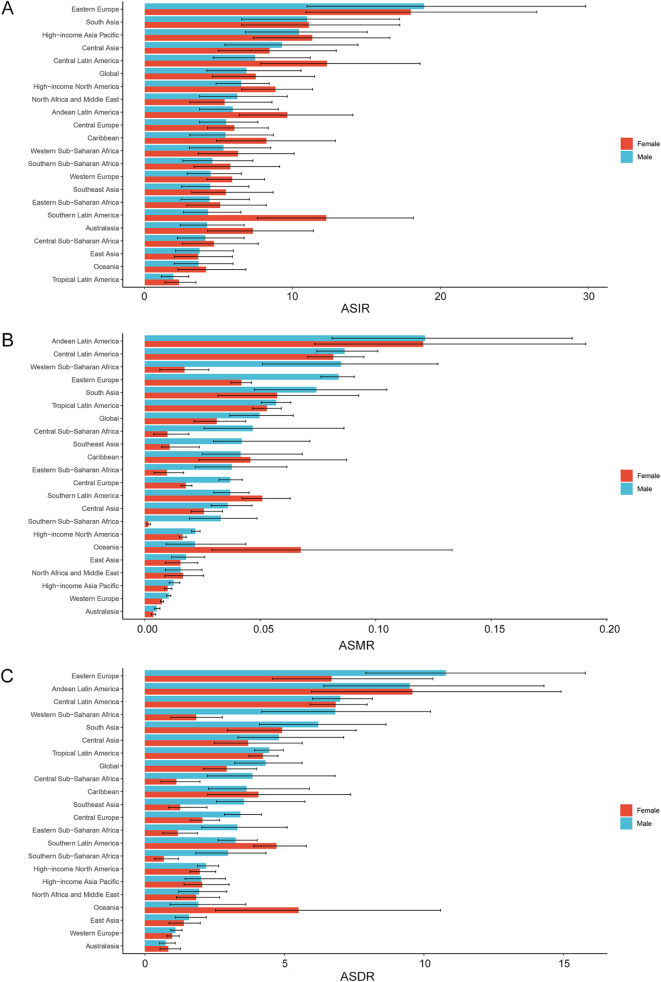
The ASIR (A), ASMR (B), and ASDR (C) for pancreatitis among children and adolescents (0–19 years) in 21 GBD regions in 2021 based on different genders. ASIR, age-standardized incidence rate; ASMR, age-standardized mortality rate; ASDR, age-standardized disability-adjusted life-year rate; GBD, Global Burden of Diseases.

Among the 21 GBD regions, the highest ASIR of pancreatitis among children and adolescents was observed in Eastern Europe [18.46/100 000 (95% UI: 11.05–28.08)], South Asia [11.04/100 000 (95% UI: 6.57–17.24)], and High-income Asia Pacific [10.87/100 000 (95% UI: 7.08–15.74)] in 2021. From 1990 to 2021, 8 of the 21 GBD regions experienced increasing ASIR trends, with South Asia demonstrating the most rapid rise [1.01 (95% CI: 0.80–1.22)]. Conversely, the remaining 14 GBD regions exhibited declining ASIRs, with East Asia achieving the fastest reduction [−1.75 (95% CI: −2.21 to −1.28); Table 1]. The ASMR remained low across all GBD regions, with the highest values reported in Andean Latin America [0.12/100 000 (95% UI: 0.09–0.17)], Central Latin America [0.08/100 000 (95% UI: 0.07–0.10)], and South Asia [0.07/100 000 (95% UI: 0.05–0.09)] in 2021. Similarly, Andean Latin America [9.54/100 000 (95% UI: 6.81–13.28)], Eastern Europe [8.79/100 000 (95% UI: 6.34–13.01)], and Central Latin America [6.92/100 000 (95% UI: 6.06–7.90)] accounted for the top three ASDR. While most GBD regions continued to show declining ASMR and ASDR trends, Southern Sub-Saharan Africa, Tropical Latin America, and Western Sub-Saharan Africa experienced slight increases in ASMR and ASDR over the past 32 years. Notably, High-income Asia Pacific [−3.64 (95% CI: −4.01 to −3.28)] and East Asia [−3.18 (95% CI: −3.31 to −3.05)] achieved the fastest reductions in ASMR and ASDR, respectively (Table 1). As for gender disparity, females exhibited higher ASIR compared to males across most GBD regions in 2021, excluding Eastern Europe, Central Asia, North Africa and Middle East, and East Asia (Fig. 2). Conversely, males demonstrated higher ASMR in most GBD regions in 2021, with exceptions observed in Caribbean, Southern Latin America, Oceania, and North Africa and the Middle East. Similarly, ASDR was consistently higher in males across most regions in 2021, except in Andean Latin America, Caribbean, Southern Latin America, High-income Asia Pacific, Oceania, and Australasia, where females experienced comparable or slightly elevated burdens (Fig. 2).

### National level

**Figure 3.**
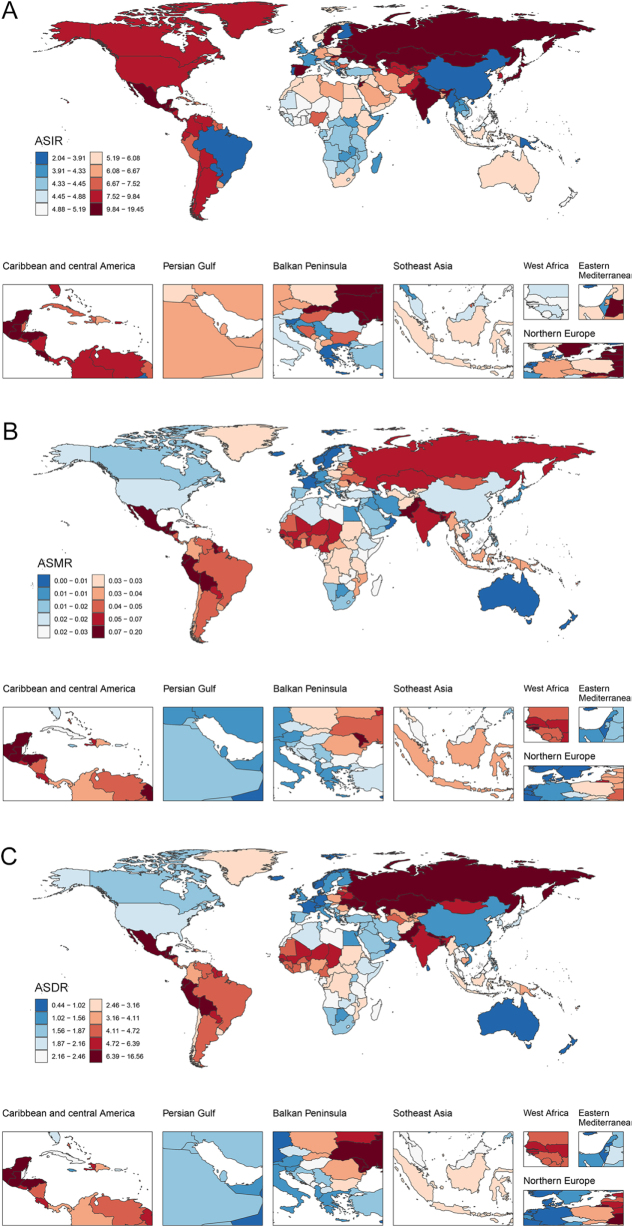
The ASIR (A), ASMR (B), and ASDR (C) for pancreatitis among children and adolescents (0–19 years) in 204 countries/territories in 2021. ASIR, age-standardized incidence rate; ASMR, age-standardized mortality rate; ASDR, age-standardized disability-adjusted life-year rate.

Nation-level disparities in pancreatitis burden among children and adolescents were observed across 204 countries/territories in 2021 (Fig. 3). India [66 722.49 (95% UI: 40 099.84–103 367.90)], China [12 202.96 (95% UI: 6782.44–20 019.47)], and Pakistan [9320.37 (95% UI: 5473.08–14 560.99)) had the highest number of incidence cases in 2021. Russian Federation [19.26/100 000 (95% UI: 11.50–29.42)], Ukraine [18.20/100 000 (95% UI: 10.81–27.80)], and Moldova [13.05/100 000 (95% UI: 7.79–19.62)] demonstrated the highest ASIR in 2021, while Tokelau [ASMR: 0.23/100 000 (95% UI: 0.11–0.48), ASDR: 18.61/100 000 (95% UI: 8.62–38.41)], Niue [ASMR: 0.20/100 000 (95% UI: 0.10–0.37), ASDR: 16.40/100 000 (95% UI: 8.50–29.55)], and Guatemala [ASMR: 0.17/100 000 (95% UI: 0.13–0.21), ASDR: 13.16/100 000 (95% UI: 10.40–16.15)] ranked highest in ASMR and ASDR (Fig. 3, Supplemental Digital Content Tables S1, S2, and S3, available at: http://links.lww.com/JS9/G611). From 1990 to 2021, 71 countries/territories saw upward trends in ASIR, with Chile achieving the fastest rise [1.78 (95% CI: 1.47–2.09)]. The ASMR decreased slightly in 157 countries/territories, while only 47 countries/territories recorded a rise in ASMR, with Georgia demonstrating the most rapid rise [6.82 (95% CI: 5.86–7.78)]. Similarly, 51 countries/territories reported rising ASDR, and Guyana saw the fastest growth [2.20 (95% CI: 1.47–2.93); Supplemental Digital Content Tables S1, S2, and S3, available at: http://links.lww.com/JS9/G611). Notably, India [deaths: 321.17 (95% UI: 191.68–459.85), DALYs: 27 737.64 (95% UI: 17 411.10–38 694.25)], Pakistan [deaths: 94.90 (95% UI: 55.09–142.88), DALYs: 7679.53 (95% UI: 4704.81–11269.23)], and Bangladesh [deaths: 77.40 (95% UI: 39.82–129.95), DALYs: 6028.80 (95% UI: 3217.06–9947.54)] experienced the highest numbers of deaths and DALYs in pediatric pancreatitis in 2021, reflecting both large population and disease burden.

### Age and sex patterns

**Figure 4.**
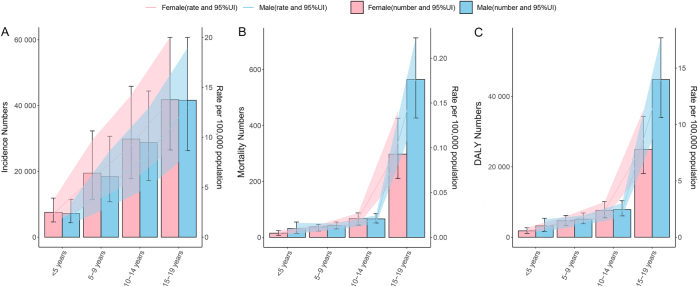
Age patterns by sex of numbers and rates of incidence (A), mortality (B), and DALYs (C) for pancreatitis among children and adolescents (0–19 years) globally in 2021. DALYs, disability-adjusted life years.

Figure 4, Supplemental Digital Content Tables S4, S5, and S6, available at: http://links.lww.com/JS9/G611, show the incidence, mortality, and DALYs of pancreatitis among children and adolescents in 2021, stratified by age and sex. Notably, females exhibited higher incidence compared to males across all age groups, whereas males demonstrated higher mortality and DALYs than females across all age groups excluding mortality in the 10–14 years age group. The pediatric pancreatitis burden grows as age increases (Fig. 4). To be specific, the incidence numbers for males and females peaked in the 15–19 years age group, with 41 622.12 (95% UI: 26 316.28–60 711.85) and 41 802.94 (95% UI: 26 502.76–60 750.89) cases, respectively. From 1990 to 2021, the incidence rate of pancreatitis decreased in children aged <5 years and 5–9 years age groups, while it increased in adolescents aged 10–14 years and 15–19 years age groups (Supplemental Digital Content Tables S4, available at: http://links.lww.com/JS9/G611). As for mortality, death numbers remained low in children <5 years [46.52 (95% UI: 25.72–72.45)], 5–9 years [79.14 (95% UI: 61.13–96.62)], and 10–14 years [132.58 (95% UI: 104.96–156.43)] age groups, whereas a higher mortality burden was recorded in adolescents aged 15–19 years [861.85 (95% UI: 701.17–1096.75)]. Among adolescents aged 15–19 years, the mortality number and rate for males [number: 564.37 (95% UI: 426.75–713.20), rate: 0.18/100 000 (95% UI: 0.13–0.22)] were nearly double that for females [number: 297.48 (95% UI: 210.72–426.27), rate: 0.10/100 000 (95% UI: 0.07–0.14)] (Supplemental Digital Content Tables S5, available at: http://links.lww.com/JS9/G611). Additionally, the DALY number and rate followed a pattern similar to that of the mortality (Supplemental Digital Content Tables S6, available at: http://links.lww.com/JS9/G611).

### Association between SDI and pancreatitis burden

**Figure 5.**
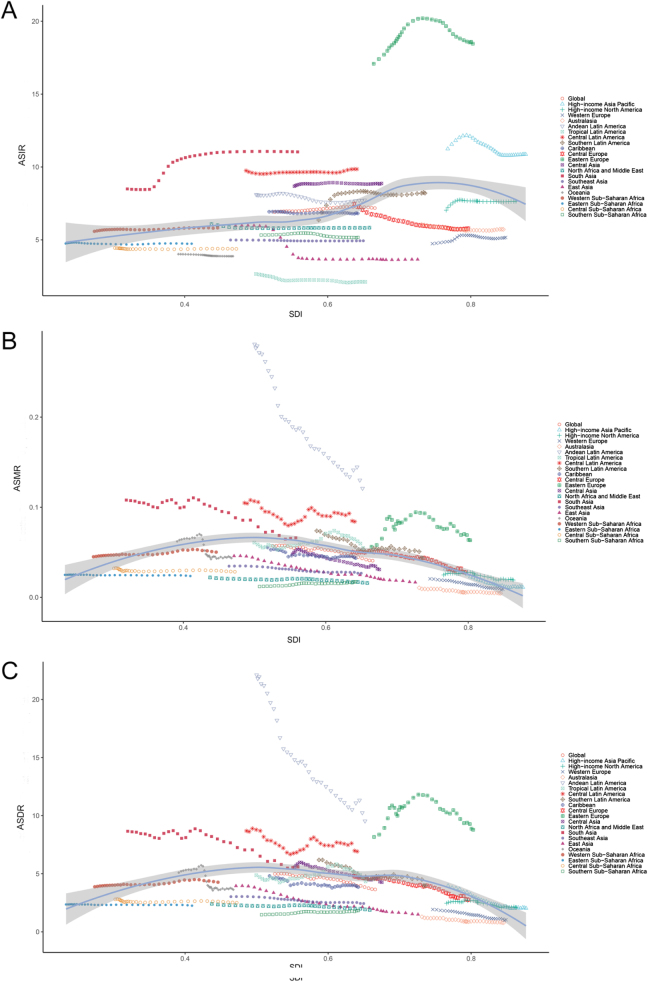
Association between SDI and ASIR (A), ASMR (B), and ASDR (C) for pancreatitis among children and adolescents (0–19 years) in 21 GBD regions between 1990 and 2021. The blue line represents expected values based on the SDI. The shaded bands around the blue line represent the 95% CI. Each point represents a region, with trends depicting the correlation between SDI and the respective rates. ASIR, age-standardized incidence rate; ASMR, age-standardized mortality rate; ASDR, age-standardized disability-adjusted life-year rate; GBD, Global Burden of Disease; SDI, sociodemographic index; CI, confidence interval.

From 1990 to 2021, significant correlations between epidemiological metrics (ASIR, ASMR, and ASDR) and SDI were observed. ASIR displayed a positive correlation with SDI (*r* = 0.3424, *P* < 0.000001), peaking at an SDI of approximately 0.75. Notable regional disparities were reported, with some regions, such as Eastern Europe, High-income Asia Pacific, and South Asia, exceeding expected ASIR values based on SDI. Conversely, some regions, such as Tropical Latin America, East Asia, and Western Europe, exhibited ASIR levels below the expected values for their respective SDI (Fig. 5A). In contrast, ASMR and ASDR showed negative correlations with SDI (*r* = −0.3417, *P* < 0.000001; *r* = −0.2516, *P* = 1.502e-11), characterized by inverted U-shaped distributions. ASMR and ASDR peaked at moderate SDI levels and were lower at both low and high SDI levels. Most GBD regions demonstrated decreasing trends in ASMR and ASDR as SDI increased over the 1990–2021 period, with Andean Latin America experiencing the largest reduction. The ASMR and ASDR of Eastern Europe initially increased, followed by a subsequent decline. Both Andean Latin America and Eastern Europe and some other regions, such as Central Latin America and South Asia, maintained higher than expected ASMR and ASDR (Fig. 5B,C). Additionally, we also revealed the association between SDI and ASRs across 204 countries/territories (Supplemental Digital Content Figure S1, available at: http://links.lww.com/JS9/G611).

### Decomposition analysis

**Figure 6.**
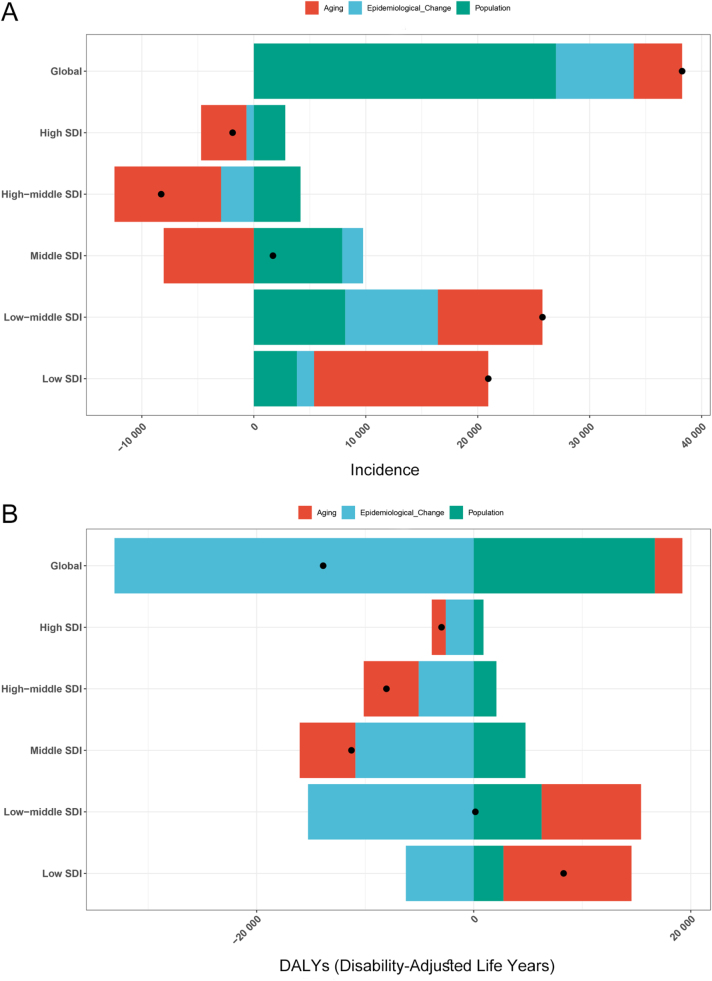
Changes in incidence (A) and DALY (B) burden of pancreatitis among children and adolescents (0–19 years) attributed to three primary factors (aging, population growth, and epidemiological change) from 1990 to 2021 at global level and SDI level. DALYs, disability-adjusted life years; SDI, sociodemographic index.

Over the past 32 years, global incidence cases of pancreatitis in children and adolescents have experienced an upward trend, with population growth (70.55%) identified as the primary driver, followed by epidemiological change (18.14%) and population aging (11.3%). While population growth emerged as the dominant factor at the global level, the influence of aging was more pronounced across all five SDI regions. Specifically, aging accounted for the largest proportional contribution to pancreatitis incidence burden in high SDI (213.84%), high-middle SDI (115.08%), middle SDI (−470.81%), low-middle SDI (36.23%), and low SDI (74.3%) regions (Fig. 6A, Supplemental Digital Content Tables S7, available at: http://links.lww.com/JS9/G611). As for DALY, epidemiological change (238.56%) was identified as the primary driver of the global DALY burden, followed by population growth (−120.28%) and population aging (−18.28%). Epidemiological change also exerted the most substantial influence across all five SDI regions with the exception of low SDI regions, and it exhibited an attenuating impact on all SDI regions (Fig. 6B, Supplemental Digital Content Tables S8, available at: http://links.lww.com/JS9/G611).

### Frontier analysis

**Figure 7.**
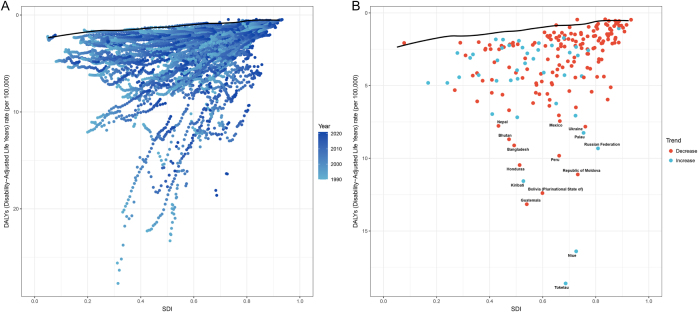
Frontier analysis of ASDR and SDI for pancreatitis among children and adolescents (0–19 years). (A) Frontier analysis based on ASDR and SDI from 1990 to 2021. (B) Frontier analysis of ASDR and SDI in 2021. The frontier line was delineated in black, with countries/territories depicted as dots. The green dots indicated an increase in ASDR for pancreatitis from 1990 to 2021, while the red dots showed a decrease. The top 15 nations with the largest effective differences were labeled in black. ASDR, age-standardized disability-adjusted life-year rate; SDI, sociodemographic index.

To evaluate potential improvement in ASDR across 204 countries/territories at different development levels, we performed frontier analysis based on ASDR and SDI from 1990 to 2021. The effective differences were mainly concentrated in nations with SDI ranging from 0.4 to 0.8, indicating a pronounced gap between observed ASDR and the achievable ideal ASDR in these countries/territories. The top 15 countries with the largest effective differences (ranging from 6.44 to 17.74) included Tokelau, Niue, Guatemala, Plurinational State of Bolivia, Kiribati, Moldova, Honduras, Peru, Russian Federation, Bangladesh, Palau, Bhutan, Ukraine, Mexico, and Nepal, representing greater unrealized opportunities for burden reduction (Fig. 7, Supplemental Digital Content Tables S9, available at: http://links.lww.com/JS9/G611).

### BAPC prediction

**Figure 8.**
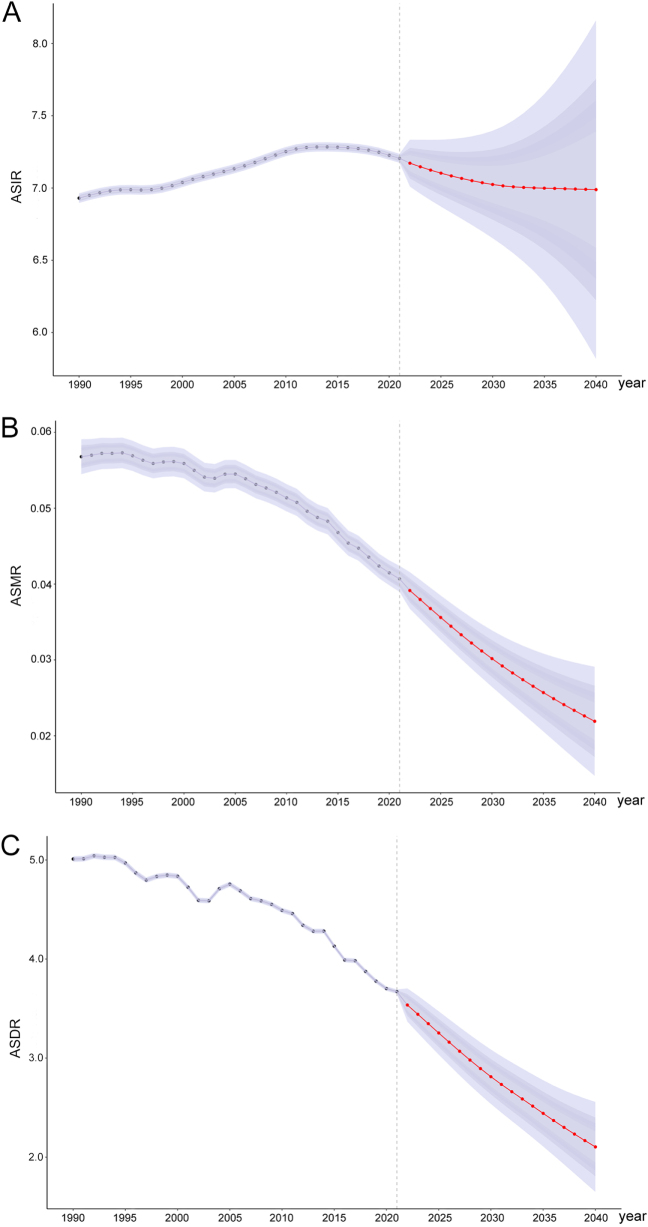
Predictions of global ASIR (A), ASMR (B), and ASDR (C) burdens for pancreatitis among children and adolescents (0–19 years) from 2022 to 2040. ASIR, age-standardized incidence rate; ASMR, age-standardized mortality rate; ASDR, age-standardized disability-adjusted life-year rate.

Based on the BAPC model, we projected the temporal trends in pancreatitis burden among children and adolescents from 2022 to 2040. The results reveal a slight decline in ASIR, followed by a stable trajectory, whereas ASMR and ASDR are expected to experience significant reductions. By the year 2040, ASIR is projected to decline to approximately 6.99 per 100 000, while ASMR and ASDR are anticipated to decrease to 0.02 and 2.10 per 100 000, respectively (Fig. 8).

## Discussion

This study employs the GBD 2021 database to systematically characterize the incidence, mortality, and DALY burden of pancreatitis in children and adolescents aged 0–19 years and identifies disparities across sex, age groups (0–4, 5–9, 10–14, 15–19 years), SDI quintiles, and countries/territories. Overall, the incidence number and ASIR of pancreatitis in children and adolescents increased globally from 1990 to 2021, while the number of mortality and DALYs decreased, as well as ASMR and ASDR. This phenomenon may be attributed to several factors. First, advancements in diagnostic modalities have substantially improved case detection rates. Previous studies have indicated that pediatric pancreatitis was historically underdiagnosed due to nonspecific clinical manifestations such as abdominal pain and vomiting, which overlap with common gastrointestinal disorders like gastroenteritis^[16]^. Unlike adults, most pediatric pancreatitis presents as mild and benign episodes. In infants, pancreatitis often presents with vague symptoms such as irritability or feeding intolerance, which are harder to identify^[17]^. The increasing utilization of sensitive imaging modalities (e.g., CT and MRI) and widespread adoption of serum biomarkers (e.g., amylase and lipase) have improved identification of mild and atypical cases^[12]^. Second, rising obesity prevalence emerges as a critical driver of pancreatitis epidemiology. Morbid obesity in pediatric AP patients is more associated with increased risks of severe AP progression, prolonged hospitalization duration, and elevated hospitalization costs compared to non-obese counterparts^[18,19]^. From 1990 to 2021, the global age-standardized obesity prevalence among school-age children and adolescents (aged 5–19 years) rose from 1.7% to 6.9% in girls and from 2.1% to 9.3% in boys, partially contributing to the observed increase in pediatric pancreatitis incidence^[20]^. Moreover, modern lifestyle transformations, involving high-sugar and high-fat diets and sedentary behaviors, lead to a rising burden of metabolic disorders such as hyperlipidemia, fatty liver disease, and type 2 diabetes in children and adolescents, which may indirectly increase pediatric pancreatitis risk^[21,22]^. Existing evidences have linked hyperlipidemia, fatty liver disease, and type 2 diabetes with AP susceptibility^[23,24]^.

As for the declining mortality and DALY burden, on the one hand, technological innovations in therapeutic modalities have improved pancreatitis management. Minimally invasive interventions, such as laparoscopic surgery and endoscopic retrograde cholangiopancreatography, have reduced surgical trauma and complications^[25]^. On the other hand, more pancreatitis cases can be detected during their initial clinical stages due to advancements in early diagnostic techniques, thereby arresting disease progression at the mild stage and improving outcomes. In contrast to adult pancreatitis, where alcohol consumption and biliary diseases are the primary etiologies^[26]^, pediatric pancreatitis demonstrates distinct etiological profiles. In children and adolescents, primary contributors include biliary disorders, medication-induced injury, congenital structural anomalies, trauma, idiopathic mechanisms, and infections^[27,28]^. Although alcoholic pancreatitis is also predominant in adolescents, its prevalence is still much lower than in adults. Since alcoholic pancreatitis is a major driver of high mortality in adults, pediatric pancreatitis mortality remains significantly lower than that of adult counterparts. Another possible reason for lower pediatric mortality is reduced prevalence of severe comorbidities in children and adolescents compared with adults, a rate reported to be less than 6%^[3]^.

The burden of pancreatitis among children and adolescents exhibits marked geographic and socioeconomic disparities across regions and countries/territories. Notably, the incidence burden is concentrated in low-middle SDI regions, attributable to socioeconomic vulnerability and limited health care accessibility. At the GBD regional level, the incidence burden is mainly concentrated in Eastern Europe, largely driven by the high ASIRs of Russian Federation and Ukraine. At the national level, Eurasian countries emerge as epidemiological hotspots, largely driven by the high ASIRs of Russian Federation and Ukraine, as well as the high incidence numbers in India and China. These findings are consistent across the three analytical levels – SDI regions, GBD regions, and national levels – with substantial overlap in the geographic regions identified at each level. The elevated burden of pancreatitis in Eastern Europe has been analyzed in several previous studies^[15,29]^, with alcohol consumption consistently identified as the primary reason. As for mortality and DALY burden, our study found that the lower the SDI, the greater the burden of pancreatitis in both mortality and DALYs. The mortality and DALY burden are concentrated in low-middle SDI and low SDI regions, characterized by limited access to advanced diagnostic and therapeutic infrastructure, insufficient supplies of essential medications, and shortages of specialized health care workers. At the GBD regional level, mortality and DALY burden are mainly concentrated in Andean Latin America, and at the national level, countries in Latin America and South Asia, as well as Russian Federation emerge as epidemiological hotspots. Latin America’s longstanding high prevalence of gallstone disease^[30]^, South Asia’s large population size^[31]^, and Russia’s high alcohol consumption patterns^[32]^ all contribute to this observed disparity.

Our research demonstrated significant age-specific and sex-related variations in the global pancreatitis disease burden among children and adolescents aged 0–19 years. Adolescents aged 15-19 years exhibited the highest incidence, mortality, and DALY burden among all four age groups (<5, 5–9, 10–14, and 15–19 years). This epidemiological pattern may be attributed to several developmental factors characteristic of late adolescence, including hormonal changes altering lipid metabolism (notably estrogen modulation in females) and increased exposure to behavioral risk factors such as consumption of high-fat diets and alcohol. Regarding gender disparities, our analysis revealed a marginally higher incidence burden in female children and adolescents than in male counterparts, probably due to the predominance of biliary pancreatitis in females compared to males^[33]^, which constitutes the most common etiology in pediatric populations. However, male children and adolescents exhibited significantly higher mortality and DALY burden than famale counterparts, possibly due to greater disease severity and complication rates^[34]^. Animal experiments showed that compared to male mice, female mice have faster onset and resolution of caerulein-induced AP^[35]^ and display lower severity of CP^[36]^, supporting our findings.

Decomposition analysis revealed that population growth emerged as the primary contributor to the increasing global incidence burden, and epidemiological changes served as the main driver of the global DALY burden. Frontier analysis highlighted considerable improvement potential in reducing pancreatitis burden within countries such as Tokelau, Niue, and Guatemala. The top 15 countries with effective differences exhibited various SDI levels (ranging from 0.43 to 0.81), demonstrating that improvement potential in pediatric pancreatitis burden does not strictly correlate with SDI levels. BAPC predictions showed a sustained decline in ASIR, ASMR, and ASDR, suggesting a favorable trajectory in disease burden reduction, highlighting the potential impact of disease management, preventive measures, and demographic changes in the future. However, predictions are based on available current data and trends, and future changes in health policies, diagnosis and treatment capacity, and risk factors may affect the actual disease burden.

We have to acknowledge some limitations in this study. First, while the GBD database aggregates multinational data, inherent variations in data completeness across countries/territories may introduce potential reporting bias. Variability in diagnostic criteria for pancreatitis across physicians and health care systems may also affect data quality. Second, the current GBD database does not stratify pancreatitis by clinical subtypes (AP, ARP, and CP), limiting our ability to conduct subtype-specific burden analyzes. Finally, risk factor data specific to pediatric populations (0–19 years) are absent, with only data on alcohol use in adolescents aged 15–19 years, precluding comprehensive etiological investigation of pancreatitis in younger age cohorts.

## Conclusions

In conclusion, our study performed a systematic and thorough assessment of the global, regional, and national burden of pancreatitis in the 0–19 years age group over the 32-year period. From 1990 to 2021, the pancreatitis incidence burden saw an upward trend, although the mortality and DALY burden declined driven by health care advancements. Low-middle SDI and low SDI regions experienced the highest pancreatitis burden, and Eastern Europe and Andean Latin America demonstrated the highest regional burdens. Adolescents aged 15–19 years require the most attention. Males bore more mortality and DALY burden than females. These findings update epidemiological data, offer guidance for resource allocation and health policy formulation, and contribute to pancreatitis management and quality of life improvement in children and adolescents.

## Data Availability

The datasets used during the current study are available from the corresponding author upon reasonable request.

## Statement

The manuscript has been read and approved by all the authors, and each author believes that the manuscript represents honest work.
